# Assessing Parents' Perception of Children's Risk for Recreational Water Illnesses

**DOI:** 10.3201/eid1105.040779

**Published:** 2005-05

**Authors:** Jacquelyn McClain, Jay M. Bernhardt, Michael J. Beach

**Affiliations:** * Council of State and Territorial Epidemiologists, Atlanta, Georgia, USA;; † Emory University, Atlanta, Georgia, USA;; ‡ Centers for Disease Control and Prevention, Atlanta, Georgia, USA

**Keywords:** Communicable Diseases, Emerging, Risk Assessment, water, Recreation, Swimming, Parent, Infant

## Abstract

Understanding people's risk perceptions and motivations to adopt preventive behavior is important in preventing the spread of recreational water illnesses (RWI) and other emerging infectious diseases. We developed a comprehensive scale measuring parents' perceived risk of their children contracting RWI. Parents (N = 263) completed a self-administered questionnaire with scale items based on 4 constructs of the Protection Motivation Theory: perceived vulnerability, perceived severity, response efficacy, and self-efficacy. Exploratory factor analysis identified 7 underlying factors, indicating 7 subscales of perceived risk for RWI. Cronbach α ranged from 0.60 to 0.81. The Precaution Adoption Process Model supported scale construct validity. This study provides the first perceived risk scale for exploring psychosocial factors that may predict or mediate the adoption of behaviors that prevent the spread of infectious diseases contracted by children while swimming. Findings from this study also provide implications for encouraging preventive behavior against other emerging infectious diseases.

Recreational water illnesses (RWI), or illnesses resulting from infectious agents acquired while swimming in pools, hot tubs, lakes, oceans, and other similar water venues, have been steadily increasing since the early 1990s, perhaps as a result of increasing numbers of bathers and the emergence of new infectious pathogens (1). Although outbreaks of RWI include a variety of illnesses, including skin, ear, eye, and respiratory infections, gastroenteritis is the most commonly reported illness (1,2). Common disinfectant agents for recreational water do not immediately destroy all pathogens, such as *Cryptosporidium parvum* (3), which pose a threat of prolonged outbreaks associated with contamination of chlorinated swimming pools (4–7).

Exposure to treated recreational water and infectious agents is high, with ≥350 million swimming visits in the United States annually (8). Children are particularly vulnerable to RWI because of their developing immune systems and high exposure to recreational water. However, many parents remain largely unaware of RWI, and most may underestimate their children's risk of getting sick from swimming (9). Preventing the spread of RWI requires a 2-fold approach with steps to prevent self-exposure and contamination of others. Swimmers must refrain from contaminating the water (e.g., avoiding swimming while having a diarrheal illness), and swimmers must also avoid exposing themselves to contaminated water, especially by swallowing it. Because parents may not perceive their children to be at risk for RWI, they have little motivation to adopt behavior modifications that can reduce the risk of their children contracting RWI and contaminating recreational water.

A person's perceived risk for an adverse outcome is considered an important factor in the adoption process of preventive behavior (10). Research on emerging infectious diseases and other health problems has found that perceived risk is an important predictor for persons taking protective actions (10–14). Perceived risk also is likely to have an important role in adopting preventive behavior against RWI (9). Although no known instrument currently exists to measure parents' perceived risk for RWI transmission to their children, having a means of gauging perceived risk is valuable for exploring how and why persons are motivated to adopt RWI preventive behavior, identifying the educational needs of a target population, and evaluating efforts designed to promote the adoption of preventive behavior.

Our perceived risk scale was informed by the Protection Motivation Theory (PMT) (15), a theory on how persons make decisions to adopt health-related behavior (16) and its 4 constructs: perceived severity and perceived vulnerability to a health threat, and response efficacy and self-efficacy to respond to the threat. Risk is generally defined as the probability of a loss or an adverse outcome and usually consists of 2 elements: the likelihood that an adverse outcome will occur and the severity of that adverse outcome (17). However, the lay public often has a more intuitive definition of risk that is based on their perceptions of the likelihood, controllability, and information available about the hazard (18). Factor-analytic research (18,19) has shown that risk perception incorporates 2 prevailing factors: 1) dread risk, which involves evaluations of control, catastrophic potential, fatal consequences, and cost-benefit ratio, and 2) unknown risk, or whether the outcome of concern is new and observable, and if its effects are immediate. The constructs of the PMT are largely consistent with the primary factors found in previous research. For example, the characteristics of dread risk are equivalent to the perceived severity and self-efficacy of the PMT, while unknown risk is similar to perceived vulnerability. PMT provides a framework to explicitly measure additional dimensions of perceived risk that are likely to predict and explain behavior related to preventing RWI. Including PMT constructs in a perceived risk scale is supported by previous research (14,20,21).

The Precaution Adoption Process Model (PAPM) (22), which describes stages of behavior adoption from being unaware of a preventive behavior (stage 1) to maintaining the health behavior (stage 7), was used to validate our scales. Because people are often motivated to adopt preventive behavior when they feel vulnerable to, threatened by, and capable of mediating a health threat (23,24), we hypothesized that respondents in stage 7 would exhibit higher mean scale scores than respondents in stage 1.

## Methods

### Sample

A convenience sample of 263 parents of children <12 years of age were recruited from 1 elementary school and 5 nonprofit recreation-focused community organizations in Atlanta, Georgia. Questionnaires were retained for analyses if the respondent indicated having at least 1 child <12 years of age and if 80% of the survey scale items were complete. Seven surveys were excluded for failing to meet these criteria, yielding 256 analyzable surveys.

Of these respondents, 213 were recruited from the community organizations and 43 from the elementary school. Respondents ranged in age from 21 to 60 years (mean 38.3, standard deviation [SD] 6.6). Most (65.6%) were female, 28.9% were male, and the sex of the rest was unknown. The ages of the respondents' children were 6 weeks to 25 years (mean 6.4, SD 4.3). Seventy-seven parents (30.1%) had children who wore diapers. Respondents' children swam frequently in chlorinated venues; more than half (61.7%) swam in chlorinated venues at least once a week.

### Procedures

After institutional review board approval was obtained from the sponsoring institution, the self-administered, anonymous, paper questionnaire was administered to parents in the fall of 2002. Parents were given verbal instructions from an oral script and asked to refer to their youngest child who swims when responding to the survey. Upon completion, respondents received an information packet on swimming safety and RWI and a $5 gift certificate.

### Instrument Development

The items included for scale development were informed from focus group findings on parents' perspectives of waterborne disease transmission in recreational water (9) and constructs from the PMT (15). Eighty-eight statements with 5-point Likert-type responses ranging from strongly disagree to strongly agree were created to reflect and capture the 4 PMT constructs. To reduce response bias, items about swimming safety were intermixed as foils. In addition, similar items were grouped by underlying construct, and items were keyed in both the positive and negative direction (25). The high internal consistency, as shown by the Cronbach α scores, indicated that keying responses in both the positive and negative direction did not compromise respondents' interpretation of questions. The questionnaire also contained a PAPM scale to stage parents on the extent to which they actively protect their children from RWI. Five statements to which respondents could either agree or disagree were used to determine respondents' stage. Before the study began, the PAPM scale was revised by 2 content experts for face validity and pilot tested among 7 parents for relevance, clarity, and readability of items.

### Data Analysis

Exploratory factor analysis was conducted by using principal axis factoring and varimax rotation. The 4 PMT constructs were factor analyzed separately. Principal axis factoring, a common factor solution that is less biased than component factor solutions because unique and error variance is eliminated from the analysis, is recommended when the factor analysis includes <12 variables (26). Varimax rotation was used to facilitate interpretability of factors by maximizing the variance of loadings on each factor (27).

Questionnaire data were included for factor analysis for a particular construct if at least 80% of all items within that construct were answered, and median replacement was used for the <1% of missing items. Variables that fell outside of the skewness range of ±2 or the kurtosis range <7 were excluded from the factor analysis. Any item within each construct that was not correlated by at least ±0.30 with at least 1 other item was eliminated from analysis. The number of analyzable cases exceeded the minimum recommended number of 5 cases per item (28) with at least 100 cases (29).

Factorability of items was confirmed by using the Bartlett test of sphericity and the Kaiser-Meyer-Olkin (KMO) measure of sampling adequacy. The number of factors extracted was determined by scree plot by using the recommended criteria (27) (i.e., eigenvalue >1 and at least 2 items loading on a theoretically interpretable factor) to yield a solution that was parsimonious yet reliable. Items with a factor loading ≥0.4 and no secondary factor loading ≥0.30 were retained (30). All emerging factors were combined to form a perceived risk of RWI scale; perceived vulnerability and perceived severity factors were combined to form a threat appraisal of RWI scale, and factors from each construct were retained as subscales. Once each scale and subscale was finalized, Cronbach a was calculated to determine scale reliability.

## Results

### Scale Development

Two hundred fifty-five cases were included in the factor analysis for perceived vulnerability items (Table 1), 1 of the 4 primary constructs informed by PMT. The KMO measure of sampling adequacy was 0.830 with a significant Bartlett test of sphericity (p<0.001). Of the 16 items entered into the factor analysis, 2 items were dropped; the 14 remaining items were in a 2-factor solution. The first factor, disease vector acknowledgment, accounted for 21.5% of the variance with an α of 0.76. This factor pertained to recognizing the swimming pool as a source of transmission of infectious agents. The second factor, knowledge of transmission of infectious agents, accounted for 7.6% of the variance with an α of 0.73. This factor referred to modes and types of diseases spread through swimming pools. These 2 factors were moderately correlated (r = 0.346, p<0.01), and combining them yielded an α of 0.79 and explained 29.1% of the variance.

**Table 1:** Perceived risk subscales and factor loadings*

Item	Factor	
	1	2
1. Disease vector acknowledgment (perceived vulnerability) (α = 0.76)†		
A well-maintained pool is germ-free.	**0.610**	0.121
Chlorinated pool water is just as clean as drinking water.	**0.607**	0.005
Chlorine kills all germs instantly.	**0.541**	0.141
A swimming pool contains fewer germs than oceans or lakes that can make my child sick.	**0.516**	0.178
My child is more likely to get sick from germs in a restaurant than from a swimming pool.	**0.501**	0.173
Pool management makes sure that the pool my child swims in is germ-free.	**0.483**	0.007
My child is more likely to get sick from germs from a public restroom than a swimming pool.	**0.459**	0.009
Swimming in chlorinated water with other swimmers can spread germs.	**0.404**	0.271
2. Knowledge of germ transmission (perceived vulnerability) (α = 0.73)†		
It is possible that there are germs in a pool that can cause eye infections.	0.140	**0.698**
It is possible that there are germs in a pool that cause skin infections.	0.211	**0.653**
Swallowing water while swimming in a pool increases the risk of getting sick from germs.	0.165	**0.586**
My child can get sick if she or he swims in a pool when another swimmer has diarrhea.	0.009	**0.492**
It is possible that there are germs in a pool that cause ear infections.	0.120	**0.485**
If one child in my family were to get sick with diarrhea from swimming in a chlorinated pool, she or he could infect the rest of the family.	0.004	**0.423**
3. Perceived severity of diarrheal illness (α = 0.65)‡		
Diarrhea is dangerous to my child's health.	**0.725**	0.190
Diarrhea threatens a child's health.	**0.611**	0.150
It is difficult for children to get well from diarrhea.	**0.422**	0.185
Compared to other children, diarrhea is more dangerous to my child's health.	**0.416**	0.003
4. Perceived severity of nongastrointestinal illness (α = 0.63)‡		
An eye infection from a germ in the pool is easily treated.	0.161	**0.739**
Children recover easily from earaches caused by germs in a chlorinated pool.	0.007	**0.604**
I am not worried about skin rashes that are caused by germs in the pool.	0.249	**0.441**
5. Response efficacy of behavioral modifications (α = 0.70)§		
Taking children on frequent bathroom breaks will reduce the feces in the pool.	**0.759**	0.101
Taking children on frequent bathroom breaks will reduce the amount of urine that will get into the pool.	**0.690**	–0.008
If parents keep their children who are sick with diarrhea out of the pool, illness to other children will be reduced.	**0.623**	0.002
Maintaining chlorine levels will reduce the number of germs in the pool.	**0.488**	0.008
Parents who avoid changing diapers near the pool help keep germs out of the pool.	**0.409**	0.103
6. Response efficacy of swim diapers (α = 0.78)§		
Swim diapers are effective in preventing feces from getting into the pool.	0.003	**0.812**
Swim diapers prevent germs from spreading in a pool.	0.129	**0.796**
7. Self-efficacy for gastrointestinal RWI prevention (α = 0.60)¶		
It is difficult to interrupt my child for bathroom breaks while she or he is playing in the pool.	**0.632**	
It would be difficult to stop my child from swimming for 2 weeks after his or her diarrhea stops.	**0.524**	
It is difficult to tell my child that she or he cannot swim when she or he has diarrhea.	**0.523**	
It is difficult to constantly supervise my children while they are playing in the pool.	**0.465**	

*Bold numbers indicate the factors on which the items load. RWI, recreational water illness. †Scales 1 and 2 combined: total variance 29.1%, α 0.79. ‡Scales 3 and 4 combined: total variance 36.8%, α 0.69. §Scales 5 and 6 combined: total variance 45.6%, α 0.63. ¶Scale 7: total variance 38.2%.

Two hundred forty-five cases were included in the perceived severity factor analysis (Table 1). The KMO measure of sampling adequacy was 0.762 with a significant Bartlett test of sphericity (p<0.001). A 2-factor solution emerged, retaining 7 of the 8 items. The first factor, severity of diarrheal illness, accounted for 28.1% of the variance with an α of 0.65. The second factor, severity of nongastrointestinal illness, accounted for 8.7% of the variance with an α of 0.63. These factors assessed perceptions of child illness severity for the most common illness (diarrhea) and other illness from RWI. These factors were moderately correlated (r = 0.316, p<0.001), and the combined a was 0.69, with 36.8% of the variance explained.

The response efficacy factor analysis included 247 cases (Table 1). The KMO measure of sampling adequacy was 0.680 with a significant Bartlett test of sphericity (p<0.001). Seven items were retained on a 2-factor solution, yielding 2 response efficacy subscales. The first factor, efficacy of behavioral modifications, accounted for 27.7% of the variance with an α of 0.71, and related to steps parents can take to reduce infectious agents in a pool. The second factor, efficacy of swim diapers, an important means of keeping fecal matter out of recreational water, accounted for 17.9% of the variance with an α of 0.78. Both subscales combined yielded an α of 0.63 with 45.6% of variance explained.

The factor analysis for self-efficacy items used 213 cases because items marked as not applicable were excluded from analysis (Table 1). The KMO measure of sampling adequacy was 0.668 with a significant Bartlett test of sphericity (p<0.001). Four items were retained in a 1-factor solution, self-efficacy for gastrointestinal RWI prevention, that explained 29.1% of the variance with an α of 0.60.

The 4 perceived vulnerability and perceived severity subscales were combined to form a threat appraisal of RWI scale with an α of 0.81. In addition, all 7 subscales from the 4 PMT constructs were combined to form a comprehensive perceived risk of RWI scale with an overall α of 0.74.

### Construct Validity

The mean scores on the 7-risk perception subscales were compared for respondents in stage 1 and stage 7 of the PAPM (Table 2). As hypothesized, respondents in stage 7 had significantly higher mean scores on the 2 perceived vulnerability subscales (p<0.001) and the 2 perceived severity subscales (p<0.001) than respondents in stage 1. The scale on the efficacy of swim diapers produced significant results (p = 0.049) in the opposite direction. The other efficacy scales produced nonsignificant differences, although some were in the hypothesized direction. Stage 1 and stage 7 respondents were also compared on the threat appraisal of RWI scale and the comprehensive perceived risk for RWI scale, and for both scales, parents in stage 7 scored significantly higher than parents in stage 1 (p<0.001), as hypothesized.

**Table 2:** Differences in perceived risk scales and subscales between stage 1 and stage 7*

Scale	Stage 1		Stage 7		
	Mean (SD)	n	Mean (SD)	n	F
Disease vector acknowledgment	23.08 (2.47)	25	27.44 (4.59)	109	21.049†
Knowledge of germ transmission	21.60 (2.96)	25	24.12 (3.18)	109	13.05†
Perceived severity of diarrheal illness	11.74 (2.56)	23	13.03 (2.51)	104	5.004‡
Perceived severity of other illnesses	8.70 (2.20)	24	10.52 (1.96)	104	15.90†
Efficacy of behavioral modifications	21.50 (2.15)	24	21.26 (2.23)	105	0.24
Efficacy of swim diapers	6.17 (2.22)	24	5.22 (2.09)	106	3.95‡
Self-efficacy for gastrointestinal RWI prevention	14.70 (3.85)	22	15.53 (2.77)	96	1.25
Threat appraisal of RWI prevention	64.78 (7.12)	23	75.29 (8.60)	104	29.76†
Perceived risk for RWI	107.11 (9.44)	18	117.52 (9.04)	90	19.62†

*RWI, water recreational disease. †p<0.001. ‡p<0.05.

## Discussion

A comprehensive perceived risk scale of RWI is an important tool for examining the psychosocial factors that predict or mediate the adoption of recommended behavior for preventing the spread of infectious diseases while swimming. This study describes the first known effort to develop a scale that offers a detailed and comprehensive assessment of parents' perceived risk for RWI for their children. The 4 components of PMT (perceived vulnerability, perceived severity, response efficacy, and self-efficacy) served as the theoretical framework for scale development, and a 7-factor solution emerged.

Factor analysis showed 2 moderately correlated factors among the perceived vulnerability items, which accounted for nearly 30% of the variance. That perceived vulnerability subscales capture hazard-related knowledge is an important attribute in forming perceived risk (31,32) because one must know how one is exposed to a hazard and the nature of the hazard to perceive being at risk. Previous research on public perceptions of food-related risks similarly found salient factors related to awareness or knowledge of food hazards (33,34).

Two moderately correlated perceived severity factors were identified that explained more than one third of the variance. A threat appraisal scale can be created by combining the perceived vulnerability and perceived severity subscales, and this scale can be useful for evaluating the impact of RWI awareness campaigns. Threat appraisal scales can assess changes in beliefs and can be effective in predicting different phases of behavior change (35).

The response efficacy subscales and the prevention self-efficacy subscale explained a great deal of variance (45.6% and 38.2%, respectively) but produced a slightly lower a when combined than when considered separately. We found that the perceived vulnerability and response efficacy subscales had sufficient (α>0.70) internal consistency (26), as did the combined threat appraisal and comprehensive perceived risk scales, but the internal consistency of the perceived severity and self-efficacy subscales were slightly lower (from 0.60 to 0.65).

Construct validation of the scales using the PAPM showed that differences in the perceived risk for RWI scale, as well as the perceived vulnerability and perceived severity subscales, were significant in the hypothesized directions, with respondents in stage 7 exhibiting lower mean scores that respondents in lower stages. However, opposite of the hypothesized direction, a significant difference in the efficacy of the swim diapers subscale was found. One explanation for this finding is that parents who are most actively engaged in preventing RWI (stage 7) may already recognize that swim diapers are not efficacious at preventing leakage of fecal matter that can contain infectious pathogens (3). Although the other response efficacy and self-efficacy subscales were not significantly different for respondents in stages 1 and 7, the difference in the self-efficacy for gastrointestinal RWI prevention was in the hypothesized direction. The lack of significant differences on these efficacy scales may be due to low levels of awareness about RWI prevention among parents, which leads to more variability on the threat scales and less on the efficacy scales.

Because a person's individual perception of risk can be influenced by a number of biases, such as personal experience and information from the media, public health practitioners need instruments to accurately assess risk perception of pediatric RWI. The scale we developed quantifies the multiple dimensions that can contribute to risk perception. This scale can be used to understand the public's perceived risk of pediatric RWI by obtaining a baseline measurement of risk perception and its contributing factors, which can inform the extent and type of educational efforts. Furthermore, scale scores can identify groups for intervention, such as those who underestimate and those who overestimate the risk for RWI. Intervention is important for these groups because an underestimation of risk will result in persons being unprepared to handle a health threat, and an overestimation of risk can result in public panic, distrust of authority, and the adoption of counterproductive behavior (36).

Factors to be emphasized in a program that aims to reduce pediatric RWI will depend on the awareness level of the targeted audience. For example, if an audience is relatively unaware of RWI, perceived vulnerability and perceived severity factors are likely to be most influential in raising risk perception. Once the risk for RWI has been acknowledged, the response efficacy and self-efficacy constructs may be more important in promoting the adoption of recommended preventive behavior modifications.

Our study had several limitations. We used a convenience sample, as is common for developing scales. Our sample was largely well-educated and consisted primarily of above-average income earners. In addition, recruitment occurred at locations where RWI awareness may be higher than average. Additional testing should involve larger and more diverse populations. In addition, the questionnaire length may have contributed to response bias, particularly toward the end of the questionnaire. Third, some respondents' self-reported RWI answers may have been influenced by social desirability bias; however, the inclusion of pool-based injury items was intended to reduce the focus on RWI.

In addition to expanding our understanding of RWI risk perceptions, the scale developed in this study may provide insights for studying how people understand and adopt preventive behavior for other emerging infectious diseases. For example, risk perception has been shown to be an important factor in obtaining vaccine for influenza (37), which can lead to serious illness in vulnerable populations, including young children (38). While vaccination is considered to be the best protection against influenza, information presented by the media might exaggerate the risks of vaccination, and the benefits of vaccine, i.e., disease prevention, are either undervalued or ignored, leading some parents to perceive vaccines to be risky (39,40). With future research, the perceived risk of the RWI scale developed in this study can be adapted to other populations, disease vectors, and pathogens, and may be useful in preventing and controlling future outbreaks.
